# TAT-IL-24-KDEL-induced apoptosis is inhibited by survivin but restored by the small molecular survivin inhibitor, YM155, in cancer cells

**DOI:** 10.18632/oncotarget.9458

**Published:** 2016-05-18

**Authors:** Jian Zhang, Rui Xu, Xinyi Tao, Yuguo Dong, Xinxin Lv, Aiyou Sun, Dongzhi Wei

**Affiliations:** ^1^ State Key Laboratory of Bioreactor Engineering, New World Institute of Biotechnology, East China University of Science and Technology, Shanghai, 200237, People's Republic of China

**Keywords:** IL-24, ER stress, apoptosis, survivin, combination therapy

## Abstract

Interleukin-24 (IL-24) is a cytokine belonging to the IL-10 gene family. This cytokine selectively induces apoptosis in cancer cells, without harming normal cells, through a mechanism involving endoplasmic reticulum (ER) stress response. TAT-IL-24-KDEL is a fusion protein that efficiently enters the tumor cells and locates in the ER. Here we report that TAT-IL-24-KDEL induced apoptosis in human cancer cells, mediated by the ER stress cell death pathway. This process was accompanied by the inhibition of the transcription of an antiapoptotic protein, survivin. The forced expression of survivin partially protected cancer cells from the induction of apoptosis by TAT-IL-24-KDEL, increased their clonogenic survival, and attenuated TAT-IL-24-KDEL-induced activation of caspase-3/7. RNA interference of survivin markedly sensitized the transformed cells to TAT-IL-24-KDEL. Survivin was expressed at higher levels among isolated clones that resistant to TAT-IL-24-KDEL. These observations show the important role of survivin in attenuating cancer-specific apoptosis induced by TAT-IL-24-KDEL. The pharmacological inhibition of survivin expression by a selective small-molecule survivin suppressant YM155 synergistically sensitized cancer cells to TAT-IL-24-KDEL-induced apoptosis *in vitro* and *in vivo*. The combined regimen caused significantly higher activation of ER stress and dysfunction of mitochondria than either treatment alone. As survivin is overexpressed in a majority of cancers, the combined TAT-IL-24-KDEL and YM155 treatment provides a promising alternative to the existing therapies.

## INTRODUCTION

Melanoma differentiation associated gene-7/interleukin-24 (mda-7/IL-24) has been initially identified by subtraction hybridization with a differentiation therapy model of human melanoma cells [1, 2]. Previous studies have demonstrated that enforced expression of IL-24 inhibits growth and promotes apoptosis in a broad range of human cancers without harming normal cells [3–6]. IL-24 can also inhibit angiogenesis, promote antitumor immune responses, sensitize cancer cells to radiotherapy-induced killing, and elicit a potent bystander antitumor activity [7–11].

Sequence analysis indicates that IL-24 is a member of the IL-10 cytokine family and it adopts an α-helical structure similar to the crystal structure of IL-10 [12, 13]. Like other IL-10 family cytokines, IL-24 binds to two type II cytokine heterodimeric receptor complexes: IL-20R1/IL-20R2 and IL-22R1/IL-20R2, and then activates the JAK/STAT signaling pathway [14–16]. Exogenous IL-24 binds with its cognate cell-surface receptors to induce apoptosis in cancer cells [17]. Studies using an adenovirus-mediated nonsecreted version of IL-24, Ad.SP-*mda-7*, have indicated that intracellular IL-24 is localized in the endoplasmic reticulum (ER) compartment. This nonsecreted protein is as effective as the full-length Ad.*mda-7* in inducing apoptosis of cancer cells [18]. More recently, it has been found that the ER-chaperone protein BiP/GRP78 is an intracellular target for IL-24. The interaction of these proteins selectively activates the ER stress-mediated cell death pathway in cancer cells [19, 20].

The transactivator of transcription (TAT) peptide of human immunodeficiency virus 1 (47–57, YGRKKRR QRRR) efficiently permeates the cytomembrane either alone or fused to proteins, DNA, RNA, or nanoparticles, even penetrating the blood-brain barrier without damage to normal cells [21–23]. The proteins resident in ER contain a C-terminal retention signal tetrapeptide KDEL (Lys-Asp-Glu-Leu). These peptides prevent the secretion of such proteins by binding with the KDEL receptors localized in the intermediate compartment and Golgi apparatus [24, 25]. In previous studies, we linked TAT and KDEL to the N-terminal and C-terminal of IL-24, respectively, and established an efficient method for obtaining recombinant TAT-IL-24-KDEL in an *Escherichia coli* expression system [26]. TAT-IL-24-KDEL has been shown to efficiently transfer into tumor cells and locate on ER, consequently inducing cell apoptosis to a much greater extent than IL-24 and TAT-IL-24.

Survivin is a member of the inhibitor of apoptosis (IAP) family of proteins. It blocks the mitochondrial pathway of apoptosis and stimulates mitosis in cancer cells [27, 28]. Survivin is highly expressed in many malignant tumors but undetectable in most corresponding normal cells [29, 30]. An increased survivin expression is associated with a poor patient prognosis and an increased rate of recurrence of various cancers [31]. Therefore, survivin has become an important biomedical target for cancer therapy. A reduction in survivin levels induces tumor cell death and makes the cells sensitive to apoptosis induced by other anticancer drugs [32]. YM155 is a novel small molecule inhibitor of survivin synthesis at the mRNA and protein levels. This molecule exhibits potent antitumor effects in a variety of human cancer cells [33]. As a result, the activation of caspases and the induction of apoptosis in hormone-refractory prostate cancer cells have been observed [34, 35].

In this study, the recombinant chimeric protein TAT-IL-24-KDEL was efficiently introduced into the ER of tumor cells; it clearly reduced the expression of survivin, which was followed by a strong induction of apoptosis. The ectopic expression of survivin prevented the TAT-IL-24-KDEL-induced reduction in survivin levels and markedly diminished TAT-IL-24-KDEL-induced apoptosis. RNA interference of survivin dramatically sensitized cancer cells to TAT-IL-24-KDEL-induced toxicity. The treatment combining TAT-IL-24-KDEL and YM155 evoked a more profound growth inhibition and apoptosis induction than either agent alone *in vitro* and *in vivo*.

## RESULTS

### TAT-IL-24-KDEL entered cells with high efficiency and distributed mainly in the ER area

**Figure 1:**
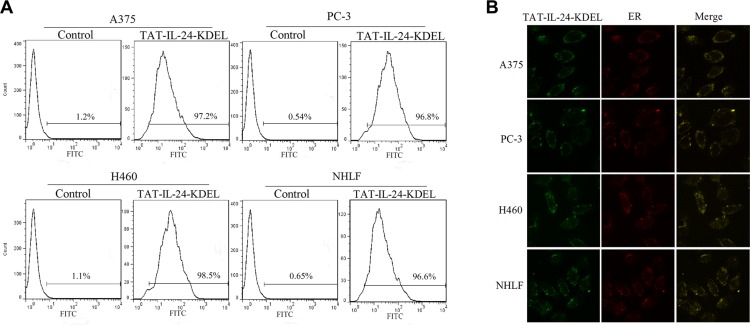
TAT-IL-24-KDEL penetrates cells (**A**) The transduction ability of TAT-IL-24-KDEL. Histogram of proteins transfection efficiency after co-cultured for 1 h with A375, PC-3, H460, and NHLF cells. All cell lines were high-efficiently transfected with TAT-IL-24-KDEL. (**B**) Intracellular distribution of TAT-IL-24-KDEL. The green color shows the fluorescein isothiocyanate-labeled proteins and the red color shows the endoplasmic reticulum (ER) stained with Texas Red (TR)-conjugated anti-calreticulin antibody. The yellow color represents the colocalization of IL-24 protein and ER (The confocal images were photographed at ×1000 magnification).

Using flow cytometry, we monitored the efficiency of TAT-IL-24-KDEL introduction into the cells of human melanoma cell line A375, human prostate cancer cell line PC-3, human NSCLC cell line H460, and the normal human lung fibroblast cell line NHLF. By FITC-labeling, TAT-IL-24-KDEL was sorted by a fluorescence-activated cell sorter (BD FACSCalibur^TM^). Cells were transfected with TAT-IL-24-KDEL with high efficiency after 1 h (Figure 1A). In A375, PC-3, H460, and NHLF cells, the transfection efficiency was 97.2%, 96.8%, 98.5%, and 96.6%, respectively.

As KDEL binds to ER retention molecules, we expected that the effect of TAT-IL-24-KDEL would be associated with ER. The FITC-labeled TAT-IL-24-KDEL was used to trace its distribution in the cells under a confocal microscope. After 12 h, the protein was located in the cytoplasm (Figure 1B). We examined the cells stained with TR-conjugated anti-calreticulin antibody and found that the TAT-IL-24-KDEL overlapped mainly with the ER area in the cytoplasm of transduced A375, PC-3, H460, and NHLF cells (Figure 1B). This property of TAT-IL-24-KDEL established a necessary foundation to locate precisely in the ER and cause the ER stress in cancer cells.

### TAT-IL-24-KDEL inhibits proliferation and induces apoptosis in cancer cells

**Figure 2:**
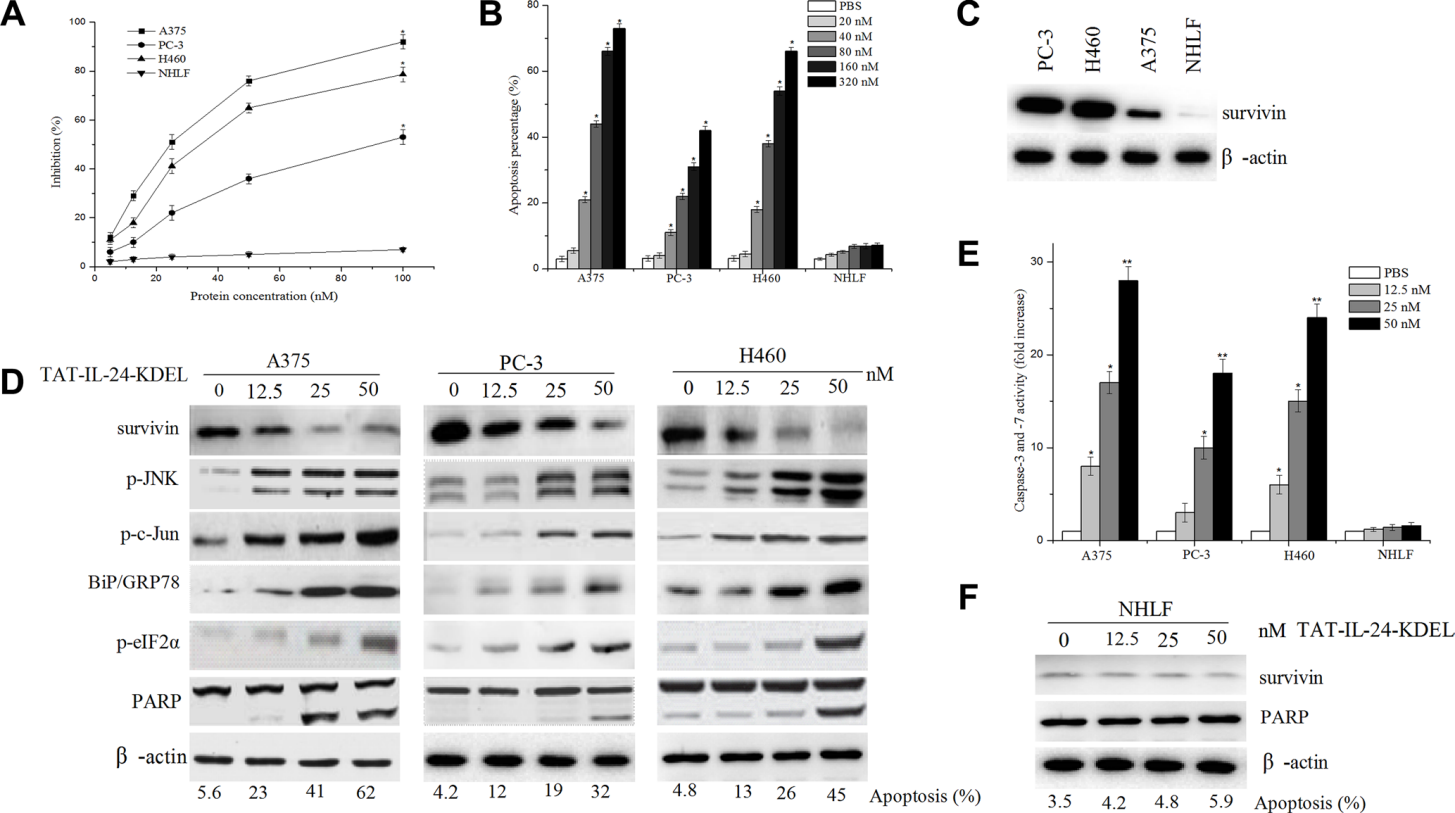
IL-24 induces apoptosis in cancer cells (**A**) Inhibition rates of four cell lines co-cultured with various concentrations of TAT-IL-24-KDEL for 72 h. (**B**) Apoptosis rate was obtained by flow cytometry analysis after cells were co-cultured with various concentrations of TAT-IL-24-KDEL for 24 h. (**C**) Cell lysates were collected from the indicated cells, and expression of survivin was determined by western blot. (**D**) A375, PC-3, and H460 cells were treated with various concentrations of TAT-IL-24-KDEL for 48 h, expression profiles of the indicated proteins were determined by western blot. (**E**) Dose response for caspase-3/7 activity in various cell lines. Cells were treated with TAT-IL-24-KDEL from 12.5 to 50 nM for 48 h. (**F**) The normal human lung fibroblast (NHLF) cells were treated with various concentrations of TAT-IL-24-KDEL, and expression of survivin and PARP was determined by western blot. Percentage of apoptosis was determined by flow cytometry. Data are presented as mean ± SD (*n* = 3; *P < 0.05; **P < 0.01 versus PBS-treated group).

We investigated the suppressive effect of TAT-IL-24-KDEL in several cell lines by measuring the viability of the cells using MTT assay. As shown in Figure 2A, TAT-IL-24-KDEL suppressed the proliferation of cancer cells; the 50% inhibitory concentration for A375, PC-3, and H460 cells was 24 nM, 95 nM, and 33 nM, respectively. The proliferation of NHLF cells was not inhibited. Flow cytometry analysis showed that the apoptosis rate of various cancer cells by TAT-IL-24-KDEL increased in a dose-dependent manner. The exception was the normal cell line NHLF, which showed no obvious increase in apoptosis after the introduction of TAT-IL-24-KDEL (Figure 2B). These results suggested that TAT-IL-24-KDEL could specifically inhibit proliferation and induce apoptosis in cancer cells.

### Treatment of cancer cells with TAT-IL-24-KDEL results in decreased survivin protein levels and induction of ER stress

A low-level of survivin expression was detected in the NHLF cells, and a robust expression of survivin was found in cancer cells A375, PC-3, and H460 (Figure 2C). The treatment of cancer cells with TAT-IL-24-KDEL resulted in a dose-dependent decrease in the survivin protein levels. These changes correlated with an increase in apoptosis (Figure 2D). When survivin was nearly extinguished, 45% of H460 cells were apoptotic, with accompanying PARP cleavage. We also determined the expression of key molecules involved in ER stress in A375, PC-3, and H460 cells after TAT-IL-24-KDEL treatment. The levels of BiP/GRP78, phosphorylation of eIF2α, JNK, and c-Jun increased in a concentration-dependent manner (Figure 2D). These results indicated that TAT-IL-24-KDEL induced cancer cell apoptosis via the cell death pathway mediated by ER stress [26]. In addition, the activities of caspase-3 and caspase-7 were increased in a dose-dependent manner (Figure 2E). |In NHLF cells, TAT-IL-24-KDEL treatment did not downregulate the survivin expression and did not increase apoptosis (Figure 2F).

### TAT-IL-24-KDEL downregulates survivin through inhibition of survivin transcription

**Figure 3:**
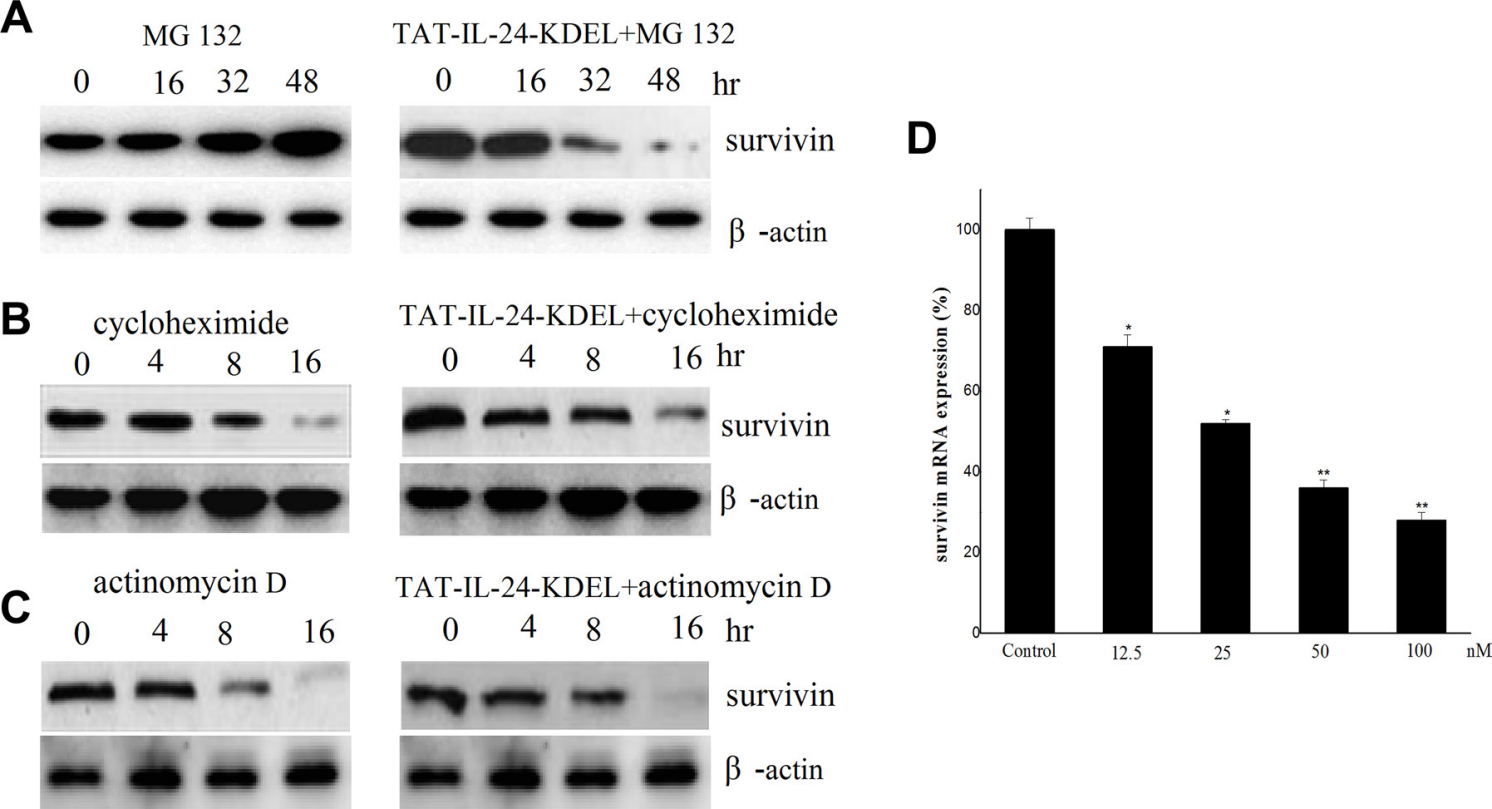
Survivin is downregulated by TAT-IL-24-KDEL at transcription level H460 cells were exposed to 1 μM MG 132 (**A**), 100 μM cycloheximide (**B**) and 1 μg/mL actinomycin D (**C**) in the presence or absence of 50 nM TAT-IL-24-KDEL for indicated periods, then cell lysates were prepared and detected against survivin antibodies. (**D**) H460 cells were treated with 50 nM TAT-IL-24-KDEL for 24 h, after that total RNA was extracted and survivin mRNA level was quantified by real-time PCR. Data are presented as mean ± SD (*n* = 3; *P < 0.05; **P < 0.01 versus PBS-treated group).

We explored the mechanism of survivin downregulation by TAT-IL-24-KDEL. H460 cells were treated with the proteasome inhibitor MG132 (1 μM) in the presence or absence of 50 nM TAT-IL-24-KDEL. TAT-IL-24-KDEL accelerated the downregulation of survivin expression. This result indicated that TAT-IL-24-KDEL inhibited survivin production at the level of transcription or translation (Figure 3A). Furthermore, H460 cells were treated with either actinomycin D (1 μg/mL) or cycloheximide (100 μM) in the presence or absence of TAT-IL-24-KDEL. TAT-IL-24-KDEL did not contribute to survivin downregulation by actinomycin D or cycloheximide, suggesting that the inhibition occurs at the transcriptional level (Figure 3B and 3C). Finally, we examined survivin mRNA using real-time PCR. TAT-IL-24-KDEL markedly decreased survivin mRNA expression in a dose-dependent manner. After treatment with TAT-IL-24-KDEL for 24 h, survivin mRNA was decreased to 36% of the mRNA in the PBS-treated control (Figure 3D).

### Survivin overexpression protects against TAT-IL-24-KDEL-induced apoptosis

**Figure 4:**
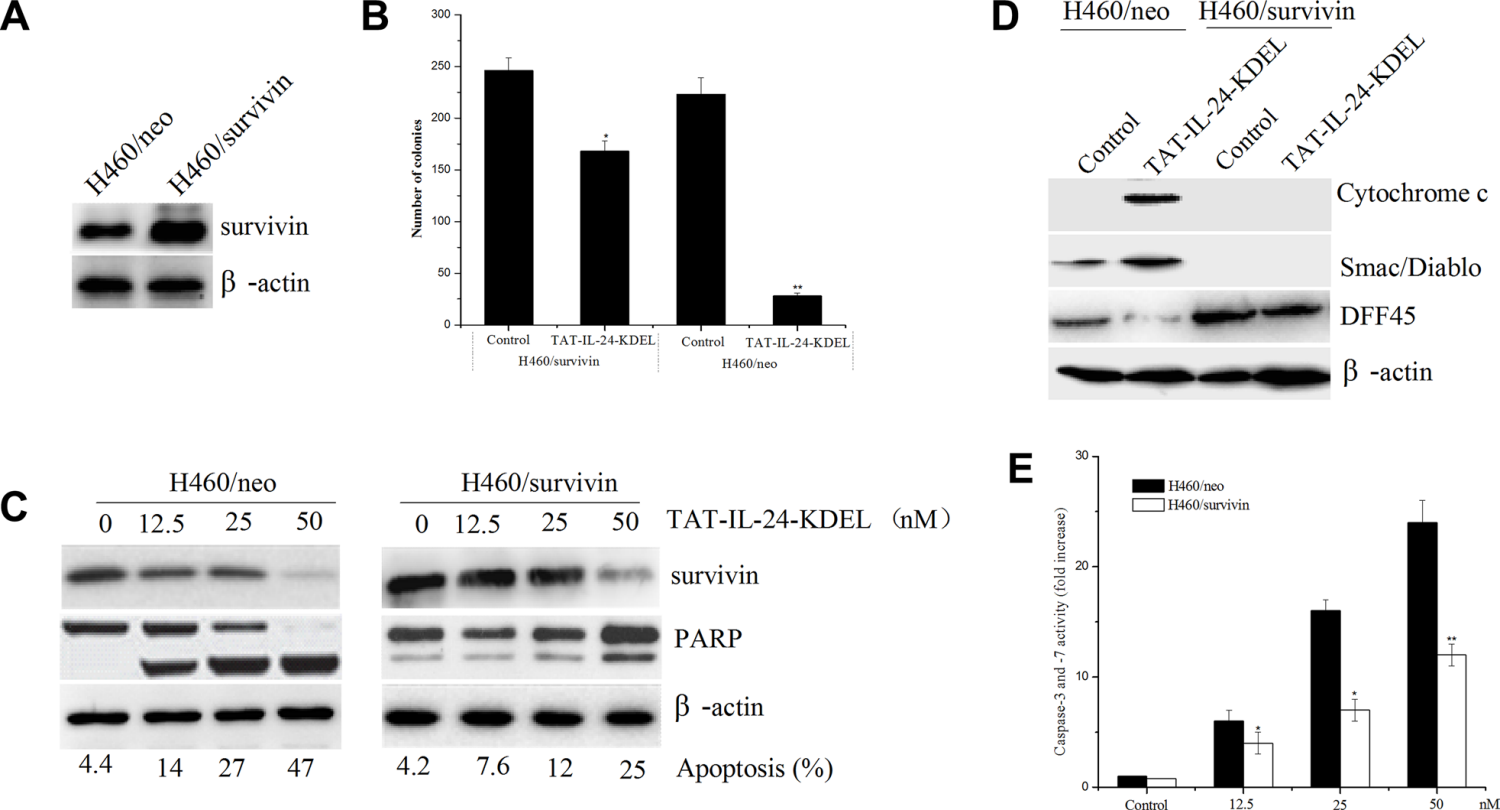
Forced expression of survivin blocks TAT-IL-24-KDEL-induced apoptosis in cancer cells (**A**) Human lung tumor cells H460 were stably transfected with the pcDNA3.1/survivin plasmid (H460/survivin) or the pcDNA3.1 vector alone (H460/neo). Survivin expression was determined by western blot. (**B**) Survivin overexpression increases clonigenic cell survival after TAT-IL-24-KDEL treatment. (**C**) Lysates were prepared from H460/survivin or H460/neo cells treated with various concentrations of TAT-IL-24-KDEL for 48 h, and western blot was performed to monitor survivin protein levels and cleavage of PARP. Percentage of apoptosis was determined by flow cytometry. (**D**) H460/neo and H460/survivin cells were treated with 50 nM TAT-IL-24-KDEL for 48 h. The cytosolic release of pro-apoptotic proteins Cytochrome *c* and Smac/Diablo was determined by western blot. The cleavage of DFF45 completely blocked in H460/survivin cells. (**E**) H460/neo and H460/survivin cells were treated with various concentrations of TAT-IL-24-KDEL for 48 h. Each bar represents the mean ± SE (*n* = 5) caspase-3/7 activity. All values were normalized to cell number and expressed as the fold increase over the control (*P < 0.05; **P < 0.01 versus PBS-treated group).

To determine the role of survivin in TAT-IL-24-KDEL-induced apoptosis, H460 cells were stably transfected with the human survivin expression construct (H460/survivin) or the vector alone (H460/neo). G418-resistant clones overexpressing survivin proteins were selected and used for subsequent experiments (Figure 4A). To determine whether survivin exerts an effect on long-term survival, we performed a colony formation assay to analyze reproductive cell death. H460/neo and H460/survivin cells were incubated in the presence or absence of 50 nM TAT-IL-24-KDEL. Treatment of H460/neo cells with TAT-IL-24-KDEL significantly abrogated colony formation compared with PBS-treated cells, whereas H460/survivin cells were protected from TAT-IL-24-KDEL-induced cell death (Figure 4B). Specifically, H460/survivin cells showed an approximately 35% reduction in clone number after TAT-IL-24-KDEL treatment compared with 88% reduction found in H460/neo cells. Furthermore, the treatment of H460/neo cells with TAT-IL-24-KDEL resulted in a marked decrease in survivin expression and induced apoptosis in a concentration-dependent manner. However, the level of survivin in H460/survivin cells remained higher than in H460/neo cells (Figure 4C). High levels of survivin significantly reduced TAT-IL-24-KDEL-induced apoptosis in the H460/survivin cells, in comparison with H460/neo cells, at all evaluated doses. In addition, a significant reduction in TAT-IL-24-KDEL-induced PARP cleavage was observed in survivin-overexpressing cells (Figure 4C). These findings indicated that survivin overexpression not only significantly extended the survival of TAT-IL-24-KDEL-treated H460 cells but also delayed cell apoptosis.

Fractionation of H460/neo and H460/survivin cells was performed, followed by an immunoblotting analysis of the cytosolic fraction. We confirmed that ectopic survivin significantly inhibited TAT-IL-24-KDEL-induced cytosolic release of pro-apoptotic proteins cytochrome C and Smac/DIABLO from mitochondria in H460/survivin cells in comparison with H460/neo cells (Figure 4D). One of the mechanisms by which survivin suppresses apoptosis is by inhibiting proteolytically processed caspase-3 and caspase-7 [28]. Using a colorimetric assay, we found that TAT-IL-24-KDEL increased the activity of caspase-3/7 in H460/neo cells. However, survivin overexpression inhibited the TAT-IL-24-KDEL-induced increase in the activity of these enzymes in H460/survivin cells (Figure 4E). The activated caspase-3 cleaves and activates the DNA Fragmentation Factor (DFF); DFF induces the nuclear DNA fragmentation, triggering apoptosis [36]. TAT-IL-24-KDEL-induced cleavage of DFF45 was entirely blocked in H460/survivin cells in comparison with H460/neo cells (Figure 4D).

### RNA interference of survivin dramatically sensitizes cancer cells to TAT-IL-24-KDEL-induced toxicity

**Figure 5:**
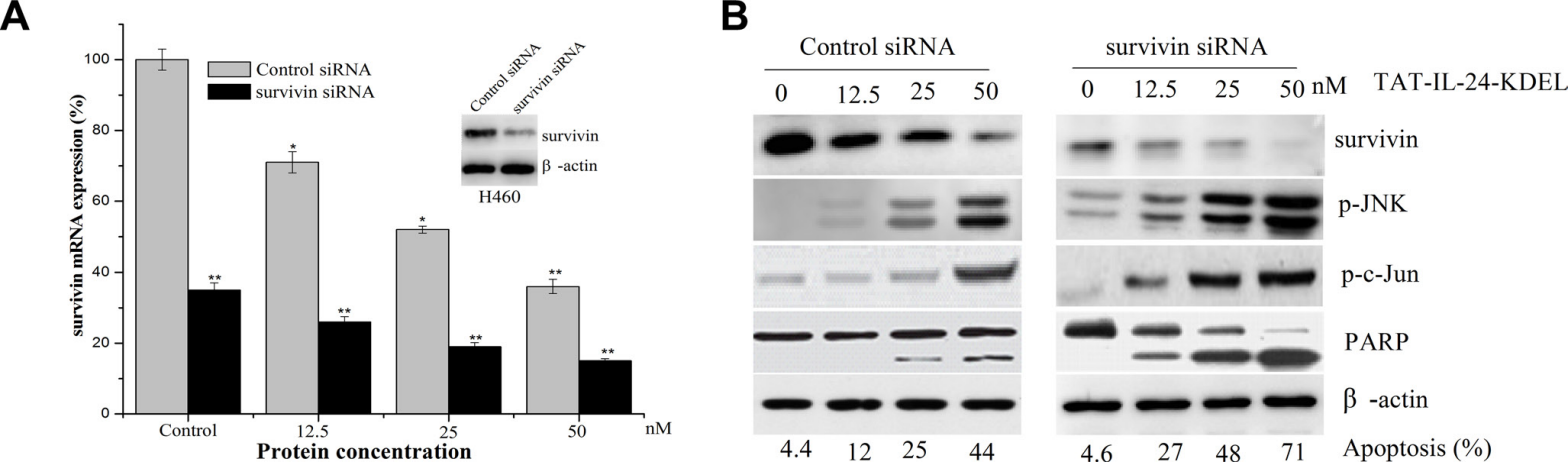
Downregulation of survivin by siRNA sensitizes cells to TAT-IL-24-KDEL-induced death (**A**) H460 cells were transiently transfected with either control siRNA or survivin siRNA for 48 h (inset); cells were incubated with siRNA for 6 h and then exposed to the indicated dose of TAT-IL-24-KDEL for an additional 24 h, after that total RNA was extracted and survivin mRNA level was quantified by real-time PCR. Data are presented as mean ± SD (*n* = 3; *P < 0.05; **P < 0.01 versus control siRNA-treated group). (**B**) H460 cells were incubated with siRNA for 6 h and then exposed to the indicated dose of TAT-IL-24-KDEL for an additional 48 h, after which the expression profiles of the indicated proteins were determined by western blot.

We used siRNA strategy to downregulate the expression of survivin protein to further evaluate the role of survivin in TAT-IL-24-KDEL-induced cytotoxicity. H460 cells were transiently transfected with siRNA against survivin (Figure 5A, inset). Western blot analysis showed that survivin protein levels were reduced by more than 80% after survivin siRNA transfection in comparison with the cells transfected with control siRNA. The expression of survivin mRNA in cancer cells after survivin siRNA transfection was lower than control siRNA treated cells, and treatment with TAT-IL-24-KDEL in survivin siRNA-transfected cells further downregulated the survivin mRNA expression level (Figure 5A). This inhibition of survivin synthesis dramatically sensitized H460 cells to TAT-IL-24-KDEL-induced apoptosis in comparison with controls (Figure 5B). There was also a significant increase in TAT-IL-24-KDEL-induced JNK and c-Jun phosphorylation, PARP activation in survivin siRNA-transfected cells (Figure 5B). These findings indicate that the siRNA-induced reduction in survivin levels significantly increases the TAT-IL-24-KDEL-induced ER stress and sensitizes the cancer cells to TAT-IL-24-KDEL-induced apoptosis.

### Isolation and characterization of resistant clones from H460 cells to TAT-IL-24-KDEL

**Figure 6:**
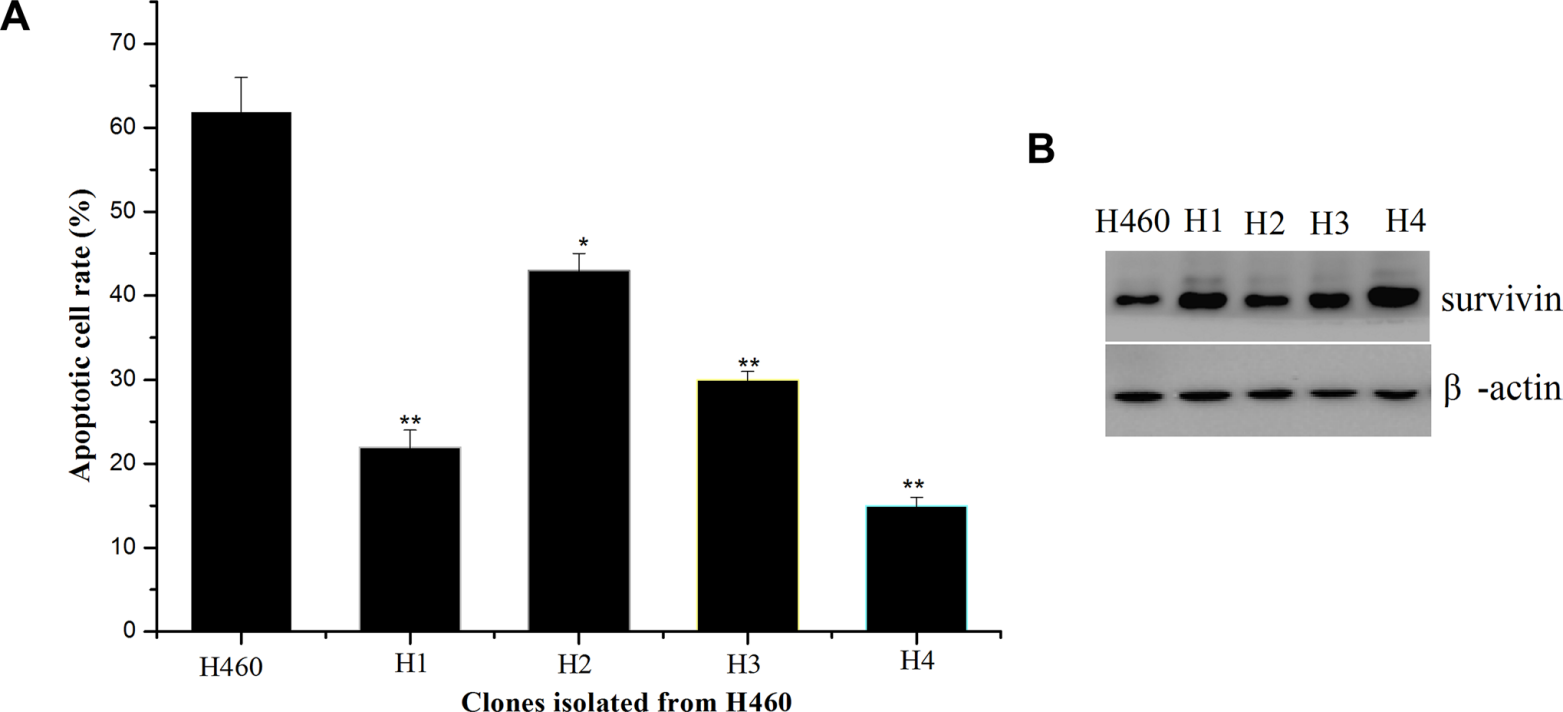
Isolation of H460 cell clones resistant to TAT-IL-24-KDEL and overexpression of survivin in the resistant clones (**A**) Rates of apoptosis induced by TAT-IL-24-KDEL (50 nM) of H460 cells and H460-derived clones (H1–H4) that were obtained after exposure to TAT-IL-24-KDEL 3 times. (**B**) Western blot analysis for survivin of H460 cells and H460-derived resistant clones (H1–H4).

To isolate resistant variants, H460 cells were exposed to 50 nM TAT-IL-24-KDEL, which induced massive cell death. The surviving cells were further cultured and re-treated with TAT-IL-24-KDEL at the same concentration. After three rounds of treatment with TAT-IL-24-KDEL and recovery, the surviving cells were seeded in 96-well culture plates for single cell cloning. The resulting 4 clones (H1-H4) showed varying sensitivity to TAT-IL-24-KDEL (Figure 6A). Since our previous studies showed that up-regulation of survivin may be responsible for conferring cancer cells resistance to TAT-IL-24-KDEL, we examined the survivin expression level of resistant clones (Figure 6B). The up-regulation of survivin level in resistant clone was consistently with the down-regulation of apoptotic rate after treated with TAT-IL-24-KDEL.

### Enhanced inhibition of *in vitro* growth by a combination of the survivin inhibitor YM155 and TAT-IL-24-KDEL

**Figure 7:**
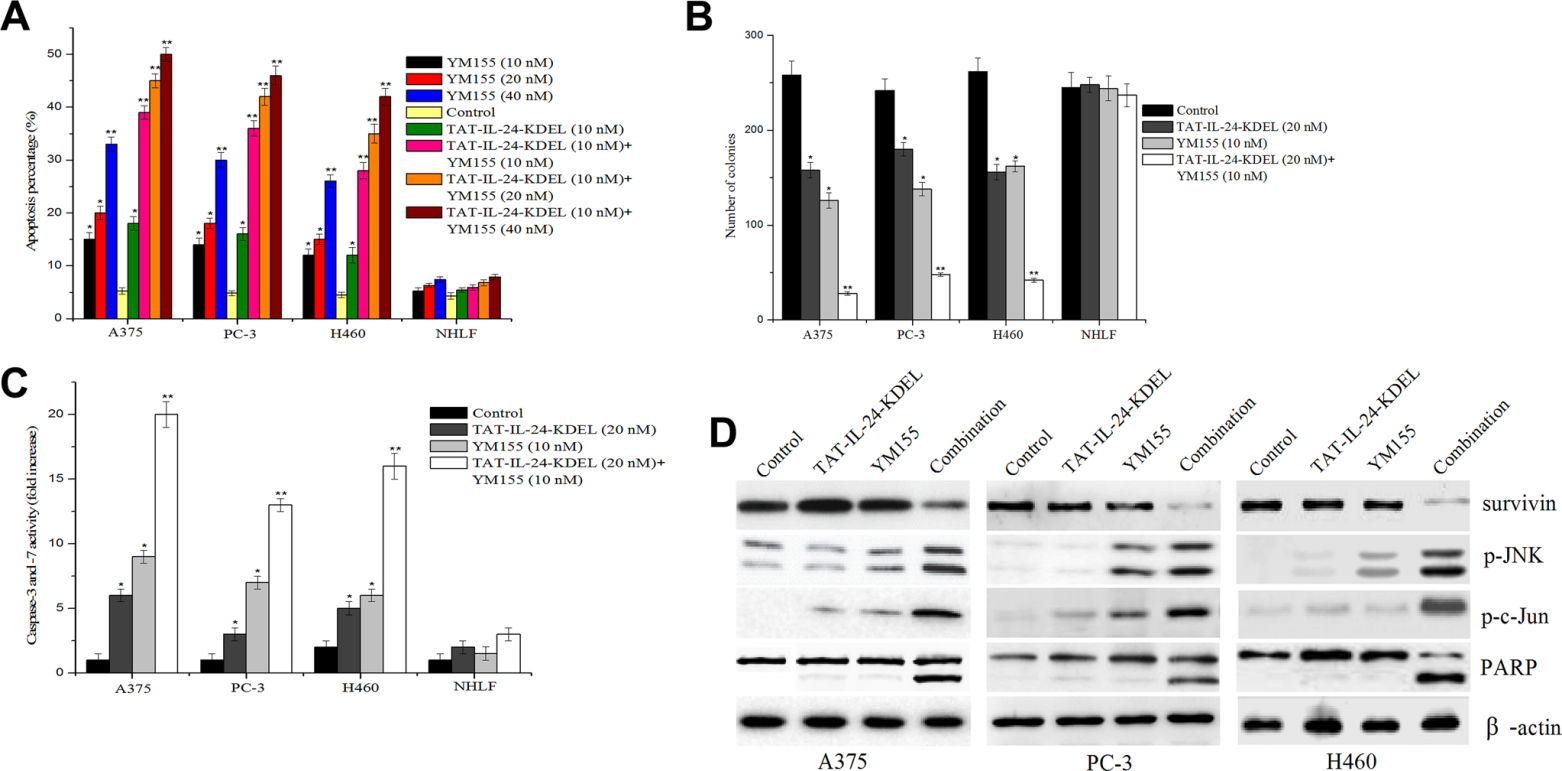
Enhanced inhibition of *in vitro* growth by a combination of YM155 and TAT-IL-24-KDEL (**A**) Cells were treated alone or in combination with varying concentrations of YM155 ranging from 10 nM to 40 nM and recombinant TAT-IL-24-KDEL at a concentration of 10 nM. Percentage of apoptosis was determined by flow cytometry. (**B**) Cells were treated either with TAT-IL-24-KDEL (10 nM) alone or in combination with survivin inhibitor YM155 (20 nM) and allowed to form colonies for 2 weeks. Colonies that contain > 50 cells were then counted. (**C**) and (**D**) A375, PC-3, and H460 cells were treated either with TAT-IL-24-KDEL (10 nM) alone or in combination with YM155 (20 nM) for 48 h. The activity of caspase-3/7 was measured and the expression profiles of the indicated proteins were determined by western blot. Data are presented as mean ± SD (*n* = 3; *P < 0.05; **P < 0.01 versus PBS-treated group).

YM155 specifically inhibits the expression of survivin and has a significant anticancer effect in preclinical models [33]. A375, PC-3, H460, and NHLF cells were submitted to the combined treatment with TAT-IL-24-KDEL and YM155 to evaluate the joint effect. In cancer cells, this treatment strongly promoted cell apoptosis in comparison with single agent treatments (Figure 7A). The combination caused greater than additive inhibition of colony formation in cancer cells (Figure 7B). In contrast, this combined treatment had little or no effect on normal NHLF cells. Furthermore, we investigated whether YM155 could potentiate TAT-IL-24-KDEL-induced apoptotic signaling. The treatment of human cancer cells with TAT-IL-24-KDEL and YM155 resulted in a synergistic phosphorylation of JNK and c-Jun, activation of PARP, and an increase in caspase-3/7 activity (Figure 7C and 7D). These results demonstrated that YM155 augmented TAT-IL-24-KDEL-induced apoptosis in cancer cells.

### Combination treatment with TAT-IL-24-KDEL and YM155 potentiates antitumor effects in nude mice model

**Figure 8:**
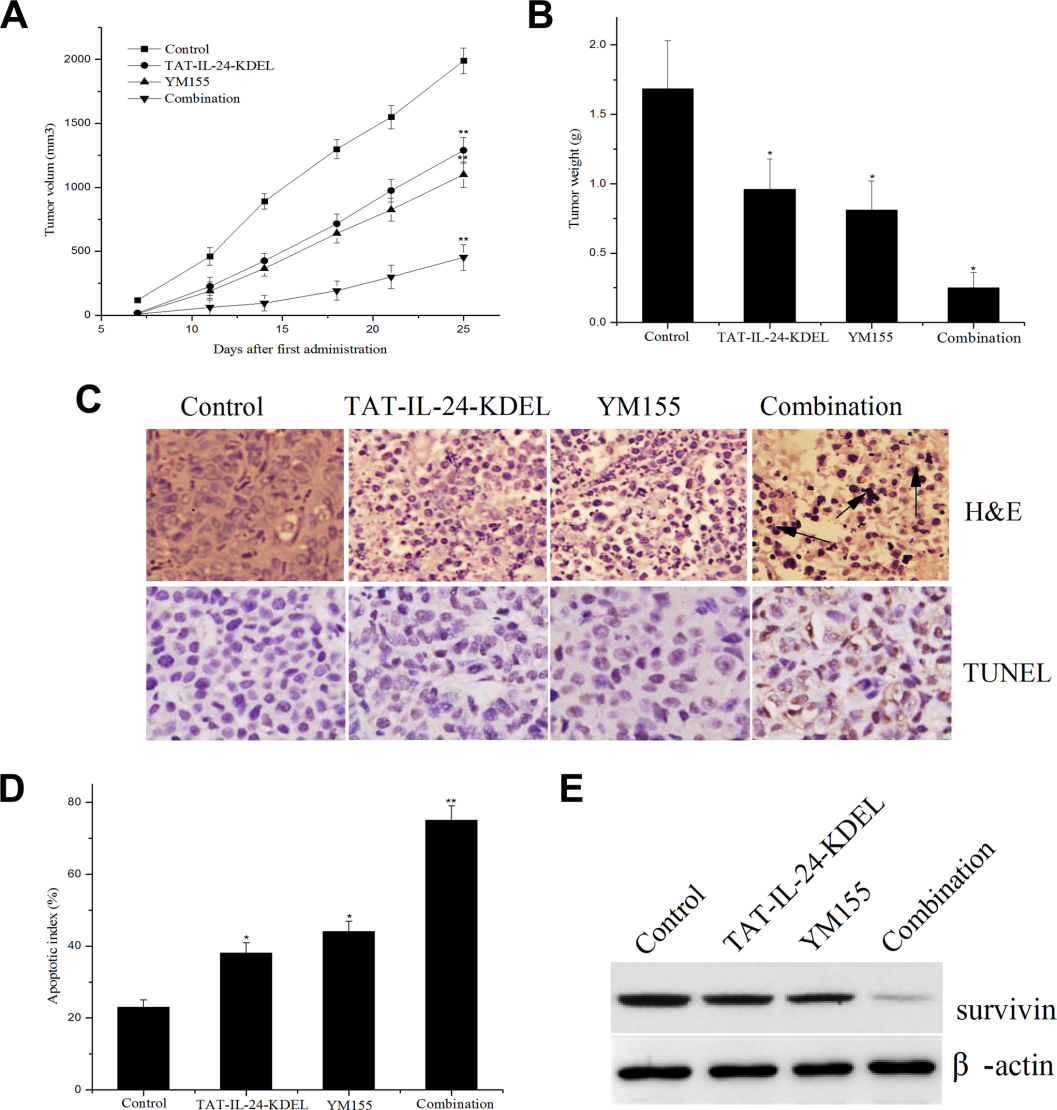
The combination regimen of TAT-IL-24-KDEL and YM155 additively inhibits tumor growth in nude mice (**A**) The combination regimen of TAT-IL-24-KDEL and YM155 additively inhibits tumor growth in nude mice. Subcutaneous xenografts of human lung cancer cells H460 in nude mice were established. The mice were treated with TAT-IL-24-KDEL (4 mg/kg) and YM155 (4 mg/kg) alone or in combination by tail vein injection (seven injections). Tumor volumes are measured twice/week. (**B**) Tumor weight of each group. (**C**) H&E and TUNEL staining of tumor tissue sections obtained from mice models with different treatments. (**D**) Quantification of percent of apoptotic index of each group. (**E**) The intratumoral survivin expression of each group. Data are presented as mean ± SD (*n* = 6; *P < 0.05; **P < 0.01 versus PBS-treated group).

To test whether the advantages of the combined *in vitro* treatment of cancer cells translates to the *in vivo* situation, we established subcutaneous xenografts of H460 cells in nude mice. After tumor size had reached approximately 100 mm^3^, the mice received tail vein injections of TAT-IL-24-KDEL (4 mg/kg) with or without YM155 (4 mg/kg) twice a week (total of seven injections each). Body weight was not markedly different between the treatment groups (data not shown), indicating the absence of systemic toxicity. The tumor volumes in the combination treatment group were much smaller than in other groups (Figure 8A). From day 12 after the first administration, the difference between the combination group and other groups became evident. On day 28, the mice were decapitated; the tumor weight in the combination treatment group was markedly lower in comparison with the single treatment groups (Figure 8B). The tumor inhibition rates of combination treatment, TAT-IL-24-KDEL, and YM155 groups were 85.1%, 42.8%, and 50.3%, respectively.

To determine the biological effect of the treatment, tumor tissue sections were subjected to H&E and TUNEL staining (Figure 8C). In the combination treatment group, there was a significant necrosis and obvious apoptosis of tumor cells. The necrosis and apoptosis were much more evident in the combination treatment group than in the TAT-IL-24-KDEL- or YM155-treated groups (Figure 8C and 8D). In addition, much more decrease in intratumoral survivin expression was observed in the combination treatment group (Figure 8E). These results demonstrated a powerful antitumor activity of the combination treatment with TAT-IL-24-KDEL and YM155 *in vivo*.

## DISCUSSION

IL-24 is a promising anticancer agent for the treatment of a wide variety of tumor cell types and has shown significant benefit in patients [37]. The replication-incompetent Ad.IL-24 inhibits cancer cell growth and induces transformed cancer cell-specific apoptosis, as well as generates antitumor responses such as antitumor immune response and the inhibition of angiogenesis [3, 7]. In addition, IL-24 is an ideal agent for combined cancer therapy. Augmented killing efficacy of IL-24 in synergy with various anticancer approaches such as chemotherapy, radiation, and monoclonal antibodies has been demonstrated in a broad range of tumor cells without harming normal cells [8, 10].

Several studies have shown that the intracellular IL-24 protein binds with BiP/GRP78, inducing ER stress selectively in cancer cells and leads to apoptosis [20]. In the present study, this recombinant chimeric protein TAT-IL-24-KDEL was efficiently penetrated into tumor cells and specifically located on the ER. The fused protein TAT-IL-24-KDEL significantly inhibited proliferation and induced apoptosis of melanoma cells A375, prostate cancer cells PC-3, and lung cancer cells H460, but did no harm to the normal NHLF cells. We found that the activation of JNK and c-Jun, the expression of BiP/GRP78, and the phosphorylation of eIF2α accompanied the TAT-IL-24-KDEL-induced cell apoptosis. Taken together, when combined with TAT and KDEL, IL-24 as a core functional domain shares the mechanisms of adenovirus-mediated intracellular IL-24; it induces cancer cell apoptosis through the ER stress-mediated cell death pathway.

Cancer cells frequently express the antiapoptotic survivin protein, which suppresses apoptosis via both intrinsic and extrinsic pathways [27, 31]. Elimination of survivin is required for apoptosis induction in response to a number of existing anticancer drugs [38, 39]. The exposure of cancer cells to TAT-IL-24-KDEL significantly downregulated survivin expression by inhibiting the survivin transcription (Figure 3). This result suggests that the survivin protein may be correlated in conferring resistance to TAT-IL-24-KDEL-induced anticancer effect. To test this hypothesis, we examined the forced expression or RNA interference of survivin in H460 cells. We found that survivin overexpression resulted in a significant reduction in TAT-IL-24-KDEL-induced PARP cleavage in the cancer cells and conferred protection against TAT-IL-24-KDEL-induced apoptosis. Furthermore, survivin overexpression inhibited the TAT-IL-24-KDEL-induced release of cytochrome *c* and Smac/DIABLO from the mitochondria. The mitochondrial survivin binds to Smac/DIABLO, delaying its release; thus, the antiapoptotic activity of survivin is neutralized [40]. The isolated resistant clones from a human lung cancer cell line H460 showed that overexpression of survivin is consistently with the acquired and inherent resistance to TAT-IL-24-KDEL (Figure 6). In contrast, RNA interference of survivin significantly reduced the expression of this protein and dramatically sensitized cancer cells to TAT-IL-24-KDEL-induced cytotoxicity. The suppression of survivin expression was accompanied by a significant increase in TAT-IL-24-KDEL-induced JNK and c-Jun phosphorylation and PARP activation. These results indicate that the reduction in survivin levels potentiates TAT-IL-24-KDEL-induced ER stress and sensitizes cancer cells to TAT-IL-24-KDEL-induced apoptosis.

YM155 is a novel imidazolium-based survivin inhibitor. This compound specifically suppressed the expression of survivin at both mRNA and protein levels. On the basis of the present data, a hypothetical model can be proposed, in which TAT-IL-24-KDEL and YM155 cooperate to induce the ER stress-mediated apoptosis. We demonstrated that a combination of TAT-IL-24-KDEL with YM155, at the doses at which single agents have little apoptotic effect, induces significant apoptosis in cancer cells and has no effect on normal NHLF cells. This combined treatment also caused a synergistic increase in TAT-IL-24-KDEL-induced apoptotic signaling involved in the ER stress. We also found that the *in vitro* effect of this treatment translates well to the *in vivo* circumstances. In our xenograft experiments in nude mice, the tumor volume and tumor weight in the combination treatment group were much smaller than in the single agent treatment groups. Histology staining detected an increase in necrosis and apoptosis in the combination treatment group in comparison with other groups. These findings suggest that YM155 has the potential to augment the therapeutic efficacy of TAT-IL-24-KDEL.

In summary, the recombinant chimeric protein TAT-IL-24-KDEL efficiently entered the tumor cells and specifically accumulated on the ER, inducing the ER stress-mediated cell apoptosis. Survivin overexpression inhibits the TAT-IL-24-KDEL-induced apoptosis at the ER level by regulating the mitochondrial apoptosis signals. RNA interference of survivin significantly potentiates TAT-IL-24-KDEL-induced ER stress and sensitizes cancer cells to TAT-IL-24-KDEL-induced apoptosis. The small molecule inhibitor of survivin, YM155, augments the therapeutic efficacy of TAT-IL-24-KDEL both *in vitro* and *in vivo*. These data provide important mechanistic insights into the relationship between survivin and IL-24 and highlight the possibility that TAT-IL-24-KDEL cooperates synergistically with YM155 to induce cancer cell death.

## MATERIALS AND METHODS

### Reagents

The PCR Purification Kit, Plasmid Mini Kit, and Gel Extraction Kit were from Promega (Madison, WI, USA). HiFiFast DNA polymerase was obtained from BiovisuaLab (Shanghai, China). Lipofectamine 2000 was from Invitrogen (Carlsbad, CA, USA). Survivin expression plasmid was constructed by the Sangon Biotech (Shanghai, China). YM155 was purchased from Selleck chemicals (Houston, TX, USA). Antibodies to IL-24, calreticulin, survivin, phosphorylated JNK and c-Jun, caspase-4, BiP/GRP78, and β-actin were obtained from Abcam (Cambridge, UK). All chemicals and reagents were purchased from Sigma unless noted specifically.

### Cell culture

Human melanoma cell line A375, human prostate cancer cell line PC-3, human nonsmall cell lung cancer cell line H460, and normal human lung fibroblast cell line NHLF were obtained from American Type Culture Collection (Rockville, MD). The cells were cultured in RPMI 1640 medium supplemented with 10% fetal bovine serum, 100 units/mL penicillin, and 100 μg/mL of streptomycin at 37°C in a 5% CO_2_ atmosphere.

### Transmembrane ability and ER localization of TAT-IL-24-KDEL

In a previous paper, we established an efficient method for obtaining recombinant TAT-IL-24-KDEL in an *Escherichia coli* expression system containing a SUMO tag [26]. The DNA sequences coding TAT and KDEL were fused with the IL-24 (GenBank No. NM_006850) at the site corresponding to its N-terminal and C-terminal portion, respectively. To obtain the recombinant protein with native N terminus, the DNA sequences of SUMO and TAT-IL-24-KDEL were fused by PCR overlap extension. Refolding of SUMO-TAT-IL-24-KDEL was performed by dialysis against refolding buffer (50 mM Tris-HCl, pH 8.0) containing 6, 4, and 2 M urea. After refolding, the His-tagged fusion protein was purified using Ni-NTA sepharose column. Eluted protein solution containing SUMO-TAT-IL-24-KDEL was incubated with SUMO protease to cleave the SUMO tag from the fusion protein. After cleavage, the mixture was passed through the Ni-NTA affinity column and the recombinant TAT-IL-24-KDEL was recovered in the flow-through fraction. To evaluate the transfection efficiency of TAT-IL-24-KDEL, fluorescein isothiocyanate (FITC) was conjugated to the fused protein for the feasibility of localization [26]. Cells were co-cultured with FITC-labeled TAT-IL-24-KDEL for 1 h, and the green fluorescence of these samples was analyzed by flow cytometer (BD FACSCalibur^TM^). The percentages of transfected cells in each sample were determined from at least 1 × 10^4^ cells.

Cells were seeded onto 35-mm plates and incubated with TAT-IL-24-KDEL (50 nM) for 12 h. Then the cells were fixed in 4% paraformaldehyde for 20 min, permeabilized with 0.2% Triton X-100 for 15 min at 20°C. These cells were stained with Texas Red (TR)-conjugated anti-calreticulin antibody for 1 h at 37°C. The cells were observed at the laser scanning confocal microscopy (Leica TCS SP2; Leica Microsystem, Wetzlar, Germany).

### *In vitro* assay for cell viability and caspase activity

Cells were seeded in 96-well culture plates at a concentration of 10^4^ cells per well. TAT-IL-24-KDEL in different concentrations was added to various cells. After incubation for 72 h, 10 μL (5 mg/mL) of MTT was added to each sample and incubated for 4 h at 37°C. The solution was discarded and 200 μL DMSO was added. After shaking gently for 10 min, the plate was read at 570 nm using microplate spectrophotometer (Bio-Rad 680; Bio-Rad, Hercules, CA). Caspase activity was determined using Caspase-Glo3/7 luminescent assay (Promega).

### Flow cytometry measurement of apoptosis

Cells were added to 6-well culture plates (6 × 10^5^ cells per well) and treated with different concentrations of TAT-IL-24-KDEL. Cells were harvested after incubation for 24 h and then incubated with the FITC-labeled Annexin-V (20 μg/mL) for 30 min to measure the early stage of apoptosis. Next, propidium iodide (50 μg/mL) was added to label the intracellular DNA. Apoptosis was then immediately quantified with flow cytometer, using FlowJo 7.6.1 software.

### Real-time PCR

Total RNA was extracted from the collected cells with Trizol reagent (Invitrogen, Carlsbad, CA, USA). Equal amounts of RNA (500 ng) from different samples were used for first-strand cDNA synthesis using SuperScript III First-Strand Synthesis System according to the manufacturer's instructions. Quantitative real-time PCR was performed in triplicate using SYBR GreenER qPCR Supermix (Invitrogen, Carlsbad, CA, USA) and the ABI 7300 real-time PCR system (Applied Biosystems, Carlsbad, CA, USA). The primers used, based on the cDNA sequences were as follows:

survivin forward: 5′-CTGCCTGGCAGCCCTTT-3′;

survivin reverse: 5′-CCTCCAAGAAGGGCCAGT

TC-3′.

GAPDH was used as a housekeeping gene to produce the normalized expression value.

### Cell transfections

Transfections were carried out using lipofectamine 2000 according to the manufacturer's instructions. Briefly, cells were seeded in 6-well plates at a density of 4 × 10^5^ cells per well and stably transfected with the human survivin expression construct (H460/survivin) or vector alone (H460/neo). Positive transfectants were selected in medium containing 400 ng/mL geneticin (G418). Cell lines were established from individual colonies using cloning cylinders. For siRNA transfection, H460 cells were transfected with survivin siRNA or control siRNA (Santa Cruz Biotechnology, Santa Cruz, CA, USA) for 48 h. After incubation, cells were harvested for western blot.

### Colony formation

The effect of TAT-IL-24-KDEL on colony formation was determined. In brief, H460/neo or H460/survivin cells were seeded into 60-mm plastic dish at a density of 200 cells per dish. TAT-IL-24-KDEL (50 nM) was added. After incubation for another 14 days, colonies were fixed with 4% phosphate-buffered formaldehyde and stained with crystal violet. Colonies that contain > 50 cells were counted.

### Western blot analysis

Cells were harvested and lysed in ice-cold RIPA buffer containing a cocktail of protease inhibitor. Equal amounts of the whole-cell lysates (40 μg per lane) were separated in 12% SDS-PAGE and transferred onto nitrocellulose membranes. The membranes were probed with the primary antibodies and the corresponding horseradish peroxidase (HRP)-conjugated secondary antibodies. Bands were visualized using an enhanced chemiluminescence detection system (Pierce^®^).

### *In vivo* antitumor activities against H460 xenograft model

All experiments that involved animals were approved by the Institutional Animal Care and Use Committee of East China University of Science and Technology and were conducted in accordance with the institutional guidelines for animal experiments. H460 cells (2 × 10^6^) were implanted into the right flanks of male athymic nude mice (16–18 g). Treatment of the tumors started when their sizes reached approximately 100 mm^3^. Each experimental group contained 6 mice. The mice were treated with TAT-IL-24-KDEL (4 mg/kg) and YM155 (4 mg/kg) alone or in combination by tail vein injection. The injections were given three times the first week and then twice/week for two more weeks for a total of seven injections. Then, the mice were further observed for another week. The weight of nude mice and tumor volumes were measured twice/week.

### H&E and TUNEL staining

After drug administration, the mice were sacrificed and sections of tumor tissue were fixed in 4% phosphate-buffered formaldehyde overnight, and stored in ethanol and embedded in paraffin. The paraffin-embedded solid tumor specimens were placed on tissue adhering slides and hematoxylin and eosin (H&E) staining was performed to detect tumor cell necrosis under light microscope equipped with a camera (Olympus BX5, Japan). The tumor tissue sections were also subjected to TUNEL assay using the *in situ* apoptosis detection kit (Promega, Madison WI, USA) according to the manufacturer's instructions. The quantification of apoptosis was determined by counting the number of apoptotic cells and dividing by the total cells in the field (> 200 cells/sample).

### Statistical analysis

Values are presented as the mean ± standard error (mean ± SE) from at least three experiments. Statistical analysis was performed by Student's *t*-test. A level of *P* < 0.05 was considered to be statistically significant.
